# New Insights in the Management of Antipsychotics in the Treatment of Schizophrenia in a Patient with Prolactinoma: A Case Report and Review of the Literature

**DOI:** 10.1155/2010/573252

**Published:** 2010-11-10

**Authors:** Elvis Henrique Santos Andrade, Pedro Mario Pan, Paula F. Ramalho da Silva, Ary Gadelha

**Affiliations:** Psychiatry Department, Federal University of São Paulo (UNIFESP), 04023-900 São Paulo, Brazil

## Abstract

Prolactinomas are the commonest pituitary adenomas and the major pathological cause of hyperprolactinaemia. Symptomatic prolactinomas are treated mainly by dopamine agonists; surgery and radiotherapy are options for nonresponders. Schizophrenia treatment is based on antipsychotics, which acts mainly at serotonergic and dopaminergic systems. We report a case of a 39-year-old schizophrenic male patient that was diagnosed with a macroprolactinoma 8 years after his first psychotic episode. The association of Schizophrenia and prolactinoma represents a clinical challenge once the treatment of one disease can exacerbate the symptoms of the other.

## 1. Introduction

Prolactinomas are adenomas originated from pituitary lactotrophs and represent about 40% of all pituitary adenomas [1]. The diagnosis is established by elevated serum prolactin levels (>100 ng/mL) and MRI imaging [1, 2]. The prolactin release is tonically inhibited by dopamine and the main clinical approach to the treatment of prolactinomas is the use of dopamine agonists, such as cabergoline and bromocriptine, which activate D2-receptors on the lactotrophs, thus promoting the reduction of tumor size [1, 2]. On the other hand, several pieces of evidence suggest that positive symptoms of schizophrenia result from a hyperactivation of dopamine transmission subcortically and the use of medications that act, among other neurotransmissions systems, at the dopamine system antagonists are the basis of its treatment [3–6]. The management of patients with both disorders is quite challenging, since the strategies of treatment confront opposing mechanisms of action.

## 2. Case Description

A 39-year-old single man was referred to the Psychiatric Department of UNIFESP by the Neuroendocrinology unit. At the age of 29, he started presenting self-reference and persecutory delusions (poisoned food, people following or accusing him), abnormal anxiety and fear of something happening to him, auditory hallucinations (familiar people asking him for help), and important impairment in social and professional performances, all of which persisted for at least 5 weeks. He was diagnosed with schizophrenia and was prescribed haloperidol 5 mg per day and biperiden 2 mg per day. Full remission was reached within a few days; however, every time the dosages were reduced, the symptoms recurred. He used both medications regularly for over 8 years and had mild impairment in global functioning. 

At the age of 37, he presented sudden bitemporal hemianopia, worse at the right side, which progressed to complete amaurosis within a month. There were no sexual complaints. Serum prolactin level was 3616 ng/mL. TSH and routine biochemical analysis were normal. An MRI scan was performed and a 25 × 20 × 12 mm pituitary macroadenoma was diagnosed. After psychiatric medication was discontinued, the patient underwent surgical intervention. The histological exam confirmed the hypothesis of macroprolactinoma. At the postoperative period, quetiapine 25 mg per day and bromocriptine 2.5 mg per day were prescribed. Quetiapine was progressively increased to 200 mg per day and later to 400 mg per day. The option for low quetiapine doses was made in order to cautiously observe serum prolactin levels. Prolactinaemia reduced to 286,7 ng/mL twenty days after the procedure.

After 6 months, serum prolactin level was 121 ng/mL and bromocriptine dosage was increased to 5 mg per day. Psychotic symptoms worsening leads to inpatient psychiatric admission. The bromocriptine dose was decreased to 1 mg per day and the quetiapine dose was increased to 600 mg per day. Stabilization in mental status was reached after four weeks. Control MRI revealed residual tumor tissue and radiotherapy was programmed with no modifications in the prescription. Patient underwent 27 radiotherapy sessions with a total dosage of 4860 cGy with no procedure-related complications. After ten months, the current serum prolactin level is 56.3 ng/mL and no positive psychotic symptoms were presented during this period. Additionally, the patient currently lives alone and does his own housekeeping. He complains of impaired initiative and lack of social interest.

## 3. Discussion

Hyperprolactinaemia usually causes oligo/amenorrhoea, galactorrhoea, and infertility in premenopausal women, and impotence, infertility, and decreased libido in men [1, 3]. Larger tumors cause compression of local structures, thus leading to hypopituitarism or neurological manifestations [1–3]. The presence of neurological symptoms, including headaches and visual impairment, ranging from quadrantanopia to bitemporal hemianopia or scotomas, are indicators of the existence of space-occupying intracranial neoplasm [1, 3–5].

Schizophrenic patients may have increased serum prolactin levels particularly due to the use of antipsychotic medication. However, serum levels superior to 100 ng/mL in women and 50 ng/ml in men, especially with concurrent symptoms, should be investigated [1, 3, 4]. In these cases, physiological (specially pregnancy) and pharmacological causes (tricyclic antidepressants, verapamil) must be ruled out first through careful clinical history [1, 3]. A complete clinical evaluation is essential, including physical examination, routine biochemical analysis (liver and renal functions), pregnancy test, and TSH serum level [1, 3]. If the patient is taking a drug known to cause hyperprolactinaemia, the drug should be withdrawn for at least 72 h, if possible [1]. An MRI of the sella turcica is indicated in the presence of neurological symptoms [1, 3]. 

Prolactinomas are the most common pathological cause of hyperprolactinaemia and are generally classified as microprolactinomas (no larger than 10 mm in diameter) or macroprolactinomas (larger than 10 mm in diameter) [1–3]. Although being almost invariably benign, occasionally they can be locally aggressive and compress contiguous structures [1–3]. The combination of hyperprolactinaemia and MRI suggestive imaging is not unequivocally diagnostic; only histopathological study is. Since the minority of the prolactinomas is removable, the strategy is to initiate dopaminergic agonist medication and consider the reduction in tumor size as confirmatory. Surgery should be a viable option if there is a good probability of removal of the entire tumor [1, 3]. Radiotherapy is reserved to refractory tumors and to cases of intolerance to dopaminergic agonists [1, 3]. 

A search at PubMed database was performed and we identified seven reported cases of prolactinoma diagnosed after the onset of a psychotic disorder [4, 5]. The use of both dopamine agonists and antagonists was inevitable and necessary to achieve clinical stability in five of them. Surgery was successful in two cases. A first generation antipsychotic accounted for psychiatric stabilization of four of these patients and quetiapine was successfully used in one case [4, 5]. 

Pal and Sarino (2000) reported a case in which a 12 mm-diameter prolactinoma was identified in a woman during a psychiatric hospitalization originally caused by acute psychotic symptoms [7]. Despite the regular daily use of 4 mg of oral risperidone and 15 mg of bromocriptine, the tumor increased and visual impairment became worse. After antipsychotic treatment was changed and olanzapine 5 mg per day was initiated, both psychotic and endocrine symptoms decreased [7]. 

Akkaya et al. (2009) reported three cases of patients diagnosed with schizophrenia [8]. Marked elevations in prolactin serum levels were identified in these patients after two weeks of use of amisulpride. Further MRI investigation revealed microprolactinomas (2 to 5 mm diameter) in all cases [8]. No prolactinoma-related intervention was performed [8].

Data from positron emission tomography (PET) studies evaluating D2 occupancy by antipsychotics indicate that thresholds of 65%, 72%, and 78% are associated with clinical efficacy, hyperprolactinaemia, and extrapyramidal symptoms, respectively [9]. First generation antipsychotics usually have higher D2 occupancy rates and have a greater affinity for tuberoinfundibular pathway [3], thus promoting higher increases in prolactin secretion [3–8]. 

Differently, second generation antipsychotics are a more heterogeneous group regarding actions on different neurotransmitters systems. Clozapine at doses of 125 to 600 mg has a D2 occupancy of 20% to 67%, below the threshold predictive of hyperprolactinaemia [9], which is consistent with the less pronounced hyperprolactinemia related to the drug [4, 9]. Thus, clozapine should be expected to have lower effect on growth of a PRL-producing tumor. Risperidone and paliperidone are associated with persistently increased prolactin serum levels, even greater than other antipsychotics [9] and, thus, should never be used in patients with prolactinomas. Olanzapine and ziprasidone are probably associated with mild-to-minimal transient elevations in prolactin levels [9]. Aripiprazol appears not to elevate prolactin levels and there are evidences that it can actually lower PRL levels at higher doses because it is a partial DA agonist [9, 10].

Quetiapine is also a second-generation antipsychotic, similar in structure to clozapine [9–11]. Clinical data indicates that quetiapine is not associated with elevations in prolactin levels [9–11]. It has been suggested that quetiapine transiently occupies D2 receptors, which allows modest occupancy of D2 receptors (0–27%) that rapidly declines to minimal levels 12 to 24 hours after the last dose [9]. Quetiapine has an overall favorable adverse effects profile and is not associated with severe complications as clozapine, such as agranulocytosis, seizures, myocarditis, and pancreatitis [9–11]. Therefore, we consider quetiapine as a first-line treatment for a psychotic patient with a concurrent prolactin-related disturbance, such as a prolactinoma. 

An *in vivo* study has shown that quinagolide crosses the blood-brain barrier to a lesser degree than bromocriptine and should, hence, be expected to exert a marked effect on the pituitary but less influence on the brain and the mental status [4]. Quinagolide is also a relatively selective D2 agonist, whereas bromocriptine has a more unselective receptor profile. Thus, it seems probable that quinagolide may be a more favorable dopamine agonist for patients with prolactinoma and psychotic disorder [4]. Nevertheless, bromocriptine was successfully used and well tolerated in five of the reviewed cases [4, 5, 8].

The clinical management of the presented comorbid affections requires special attention. A closer followup, especially in the beginning of the treatment, is mandatory, at least until the balance between prolactin serum levels and psychotic symptoms is achieved. The specific choice of both dopaminergic agonist and antagonist medications should be made individually. Selective dopaminergic agents, such as quinagolide, and antipsychotics with less action in the tuberoinfundibular pathway, such as quetiapine and clozapine, are theoretically preferable drugs. Surgery and radiotherapy are options for refractory cases.
